# Investigating the Role of Ferrous Ions in Depressing Calcite to Achieve Selective Rhodochrosite Flotation: Surface Chemistry and Experimental Insights

**DOI:** 10.3390/molecules31050896

**Published:** 2026-03-08

**Authors:** Xiao Meng, Yanhai Shao, Hongqin Chen, Xinru Jia, Hong Lin, Chengxiang Li, Jinhui Li

**Affiliations:** 1 State Key Laboratory of Complex Nonferrous Metal Resources Clean Utilization, Kunming University of Science and Technology, Kunming 650093, China; mengxiao20211202@163.com; 2 Faculty of Land Resources Engineering, Kunming University of Science and Technology, Kunming 650093, China; chq725309@163.com (H.C.); 13753493605@163.com (X.J.); 15572660706@163.com (H.L.); 15352568694@163.com (C.L.); 15096956049@163.com (J.L.); 3 Key Laboratory of Green and Intelligent Development and Efficient Utilization of Strategic Mineral Resources of Xinjiang Production and Construction Corps, School of New Energy and Mining, Xinjiang University of Technology, Hetian 848000, China

**Keywords:** rhodochrosite, calcite, flotation, ferrous ions, depressant

## Abstract

Modulating surface characteristics via metal ions has proven to be a successful approach to enhance the flotation efficiency of carbonates. Consequently, this research thoroughly examines how ferrous ions (Fe^2+^) influence the selective separation of rhodochrosite from calcite. Flotation experiments revealed that at pH 9.0, Fe^2+^ strongly depressed calcite flotation (recovery < 20%) while exerting a negligible influence on the floatability of rhodochrosite (recovery > 75%), enabling effective selective separation. To elucidate the underlying mechanism, contact angle measurements, zeta potential analysis, ToF-SIMS, SEM-EDS, XPS and Visual MINTEQ solution chemistry calculations were employed to characterize mineral surface properties. The results demonstrate that Fe^2+^ undergoes chemisorption onto the calcite surface, inducing the formation of a dense, uniform iron hydroxide layer. This layer creates a stable hydrophilic barrier that inhibits collector adsorption. In contrast, only a thin, discontinuous layer forms on the rhodochrosite surface, which is insufficient to hinder collector interaction. These findings reveal the intrinsic mechanism of selective interfacial regulation by ferrous ions, providing a new theoretical basis for the flotation separation of refractory carbonate minerals.

## 1. Introduction

Manganese is a strategic industrial metal, primarily employed in ferrous metallurgy as a deoxidizer and desulfurizer [1,2,3]. By scavenging oxygen and sulfur impurities from molten steel and controlling carbide precipitation, it significantly enhances the mechanical properties of the final product. Beyond metallurgy, manganese applications span diverse sectors, including agriculture, pharmaceuticals, food processing, electronics, and construction materials [4,5,6].

China’s manganese resources are predominantly characterized by low ore grades, high impurity levels, and complex extraction requirements. Exacerbated by a recent decline in domestic output, local supply fails to meet industrial demand, resulting in an import dependency exceeding 80% [7,8,9]. Although over 150 manganese minerals have been identified, only a select few are economically viable for large-scale exploitation. Among these, pyrolusite, psilomelane, and rhodochrosite possess primary industrial significance. Among the manganese reserves in China, rhodochrosite is the predominant mineral, occupying a proportion of over 80%. However, these ores typically exhibit low grades, severe weathering, and fine-grained dissemination, classifying them as refractory resources [10]. Given the limitations of traditional magnetic separation in recovering fine particles [11], flotation has emerged as the preferred beneficiation technique for fine-grained disseminated rhodochrosite.

Fatty acid collectors, particularly sodium oleate (NaOL), serve as the primary reagents in rhodochrosite flotation. However, despite their robust collecting efficiency, their industrial utility is often compromised by poor selectivity. The separation process is further complicated by the ubiquitous association of rhodochrosite with carbonate gangue minerals (e.g., calcite and magnesite), which possess remarkably similar surface physicochemical properties. Furthermore, metal ions (e.g., Mn^2+^, Ca^2+^, Mg^2+^, Fe^2+^, and Fe^3+^) are inevitably introduced into the flotation pulp via process water, mineral dissolution, and equipment wear. These ions alter mineral surface characteristics, thereby exerting a profound impact on flotation efficiency and selectivity [12,13,14,15,16]. Consequently, the development of efficient depressants to enhance separation selectivity remains a paramount objective in rhodochrosite beneficiation.

Metal ions such as Fe^2+^ and Fe^3+^ are frequently employed as auxiliary components within combined reagent systems. These ions modulate mineral surface properties to enhance separation efficiency. Previous studies demonstrate that introducing optimized dosages of Fe^2+^ or Fe^3+^ into sodium oleate flotation systems significantly promotes the activation and subsequent flotation of oxides including scheelite and rutile. Specifically, at pH 9.0~10.0, using a combined depressant system of sodium silicate and ferrous sulfate (4:1 mass ratio) with an oleic acid collector achieved a scheelite concentrate grade > 62% and a recovery > 89%. This performance surpassed that of sodium silicate alone [17]. Similarly, a binary system of Fe^3+^ and citric acid effectively separated scheelite from calcite. At pH 9.5 ± 0.1, with Fe^3+^ (1 × 10^−4^ mol/L) and citric acid (5 × 10^−4^ mol/L), scheelite and calcite recoveries were 81.05% and 8.95%, respectively. In contrast, citric acid alone yielded a separation efficiency (recovery difference) of only 25%, highlighting the critical role of metal ions in modulating reagent properties and mineral surface characteristics [18]. Similarly, in rutile flotation, pretreatment with Fe^3+^ and benzohydroxamic acid (BHA) significantly increased rutile recovery from 42.27% to 75.69% and expanded the optimal pH window to 4.0~8.0 [19]. Collectively, these findings demonstrate that Fe^2+^/Fe^3+^ ions function as critical auxiliary modulators within combined reagent schemes, effectively enhancing flotation separation performance.

However, existing literature largely focuses on the synergistic effects between metal ions and organic or inorganic depressants, treating metal ions primarily as auxiliary agents. The depressive efficacy, interfacial mechanisms, and mineral selectivity of metal ions acting as standalone depressants have not been systematically explored. This gap is particularly pronounced in carbonate mineral systems (e.g., calcite-rhodochrosite). For instance, in the flotation separation of fluorite from calcite, high metallurgical performance (80.29% CaF_2_ grade, 90.79% recovery) was achieved using a composite iron system (FeSO_4_/Fe_2_(SO_4_)_3_) and NaOL collector at pH 6.0 [20]. Mechanistic studies suggest that the interaction between iron salts and surface anions leads to the in situ precipitation of hydrophilic iron species (e.g., hydroxides and oxyhydroxides) on the calcite surface, which effectively inhibits NaOL adsorption. However, the complexity inherent in such composite reagent systems obscures the intrinsic contribution of the metal ions. Consequently, elucidating the selective regulatory capability of metal ions in the absence of co-depressants remains a critical scientific gap to be addressed.

In light of these challenges, the present work introduces an innovative strategy employing FeCl_2_ as the exclusive depressant to realize the efficient separation between rhodochrosite and calcite. Compared with Fe^2+^, Fe^3+^ is more prone to hydrolysis, leading to the formation of various hydroxyl complexes, e.g., Fe(OH)^2+^ and Fe(OH)_2_^+^. These species subsequently polymerize into polynuclear hydroxy complexes, eventually forming amorphous Fe(OH)_3_ precipitates. This rapid precipitation, which accelerates at pH > 2.5 [21], tends to form non-selective colloidal coatings, rendering Fe^3+^ unsuitable as a sole depressant. In contrast, Fe^2+^ is characterized by a higher hydrolysis onset pH (> 6.0) and limited hydrolysis extent, primarily existing as Fe(OH)^+^ without readily forming stable precipitates [22]. In a mildly alkaline environment, the controlled hydrolysis and oxidation of Fe^2+^ facilitate the formation of differentiated hydroxylated films on distinct carbonate mineral surfaces, thereby enhancing the potential for selective depression [16,23]. This study evaluates the efficacy of Fe^2+^ as a solitary depressant via single-mineral micro-flotation experiments. Additionally, a combination of analytical techniques—including wettability and electrokinetic tests. Furthermore, advanced surface analyses using X-ray Photoelectron Spectroscopy (XPS) coupled with Time-of-Flight Secondary Ion Mass Spectrometry (ToF-SIMS) were performed in conjunction with Visual MINTEQ simulations. These methods served to thoroughly reveal the fundamental reasons behind the alteration of calcite’s surface properties. The findings highlight an innovative pathway for the effective segregation of rhodochrosite against calcite without the need for auxiliary depressants. Moreover, this work provides a new theoretical framework for understanding how metal ions independently modulate mineral interfaces.

## 2. Results

### 2.1. Micro-Flotation Experiments

**Figure 1:**
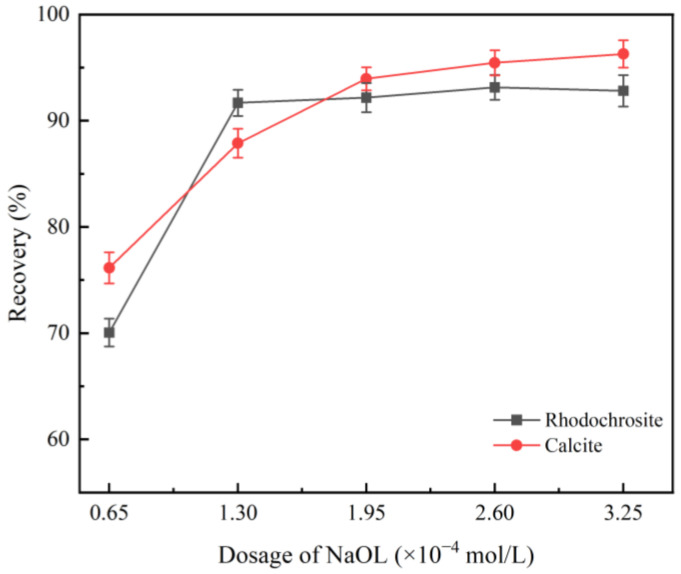
Flotation recovery of rhodochrosite and calcite as a function of NaOL concentration (pH 9.0).

Figure 1 displays the recovery trends of rhodochrosite and calcite as a function of NaOL concentration (pH 9.0, without Fe^2+^). It was observed that the recovery rates for the two minerals ascended sharply with increasing NaOL dosage up to 1.3 × 10^−4^ mol/L, maintaining a plateau thereafter. From these data, it is evident that the similarity in floatability between the two minerals makes the use of a selective depressant indispensable for their effective separation.

**Figure 2:**
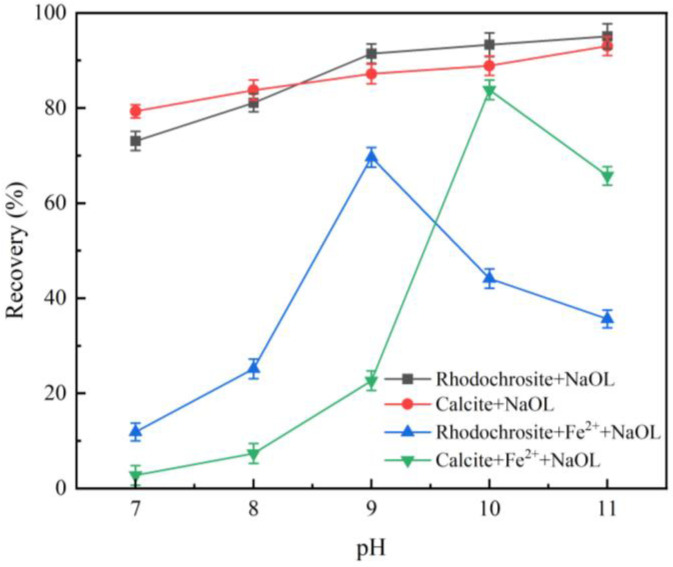
Flotation recovery of rhodochrosite and calcite as a function of pH with and without Fe^2+^ addition. (NaOL: 1.3 × 10^−4^ mol/L, Fe^2+^: 1.5 × 10^−3^ mol/L).

Figure 2 presents the effect of pH on the flotation recovery of rhodochrosite and calcite at a NaOL concentration of 1.3 × 10^−4^ mol/L and an Fe^2+^ concentration of 1.5 × 10^−3^ mol/L. Under conditions void of a depressant, the floatability of both minerals was nearly identical within the pH range of 7.0~11.0, suggesting that selective separation is unattainable. Upon adding 1.5 × 10^−3^ mol/L Fe^2+^, recoveries for both minerals initially increased and then decreased as pH rose. At pH 9.0, rhodochrosite recovery peaked at 69.64%, whereas calcite recovery dropped to 22.64%, yielding a difference of approximately 47 percentage points. Conversely, calcite reached its maximum recovery of 83.80% at pH 10.0. Thus, Fe^2+^ shows great promise for the selective inhibition of calcite at pH 9.0.

**Figure 3:**
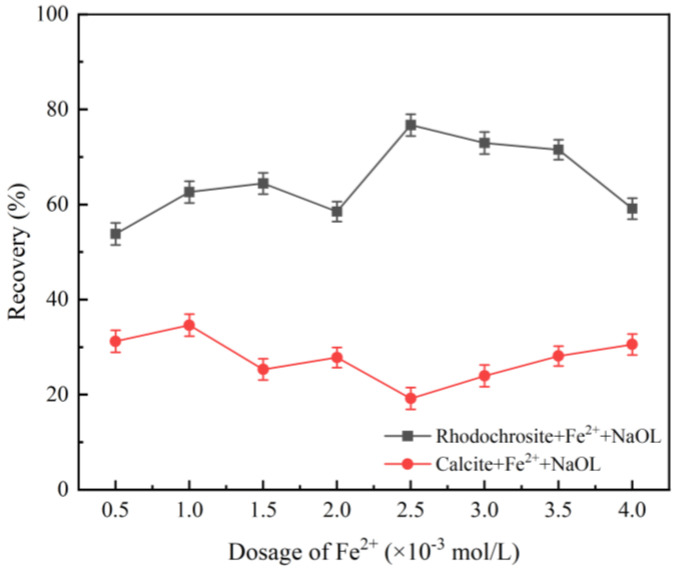
Effect of Fe^2+^ dosage on the recovery of rhodochrosite and calcite (NaOL: 1.3 × 10^−4^ mol/L, pH = 9).

Figure 3 illustrates the effect of Fe^2+^ concentration on flotation recovery at pulp pH 9.0 and a sodium oleate (NaOL) dosage of 1.3 × 10^−4^ mol/L. Optimal selectivity was observed at an Fe^2+^ concentration of 2.5 × 10^−3^ mol/L, where the recovery difference peaked. At this dosage, rhodochrosite and calcite recoveries were 76.71% and 19.19%, respectively. When the Fe^2+^ concentration exceeded this optimum, rhodochrosite recovery decreased slightly, whereas calcite recovery gradually increased. Consequently, 2.5 × 10^−3^ mol/L was identified as the optimal concentration for the effective separation of the two minerals.

### 2.2. Contact Angle Measurements

**Figure 4:**
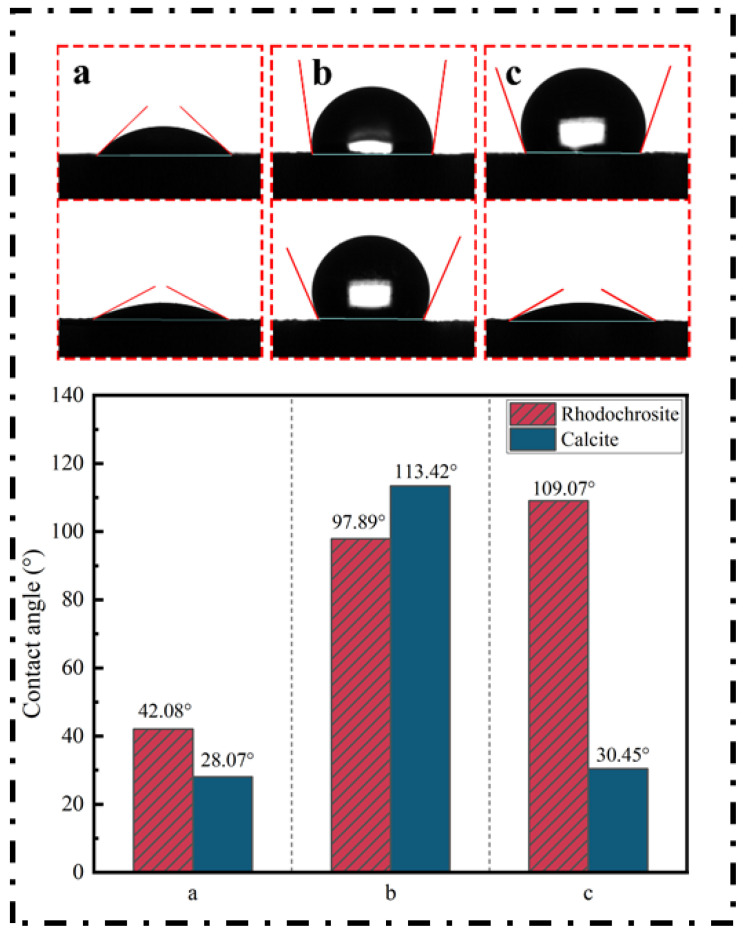
Detection of mineral surface hydrophobicity before and after Fe^2+^ addition. (**a**) Rhodochrosite/Calcite. (**b**) Rhodochrosite + NaOL/Calcite + NaOL. (**c**) Rhodochrosite + Fe^2+^ + NaOL/Calcite + Fe^2+^ + NaOL).

To assess changes in surface wettability, contact angle analysis was performed, with the results at pH 9.0 presented in Figure 4. For the bare mineral surfaces, the recorded angles were 42.08° for rhodochrosite and 28.07° for calcite. Upon conditioning with NaOL (Figure 4b), these values surged to 97.89° and 113.42°, respectively. This dramatic rise confirms that NaOL adsorbs strongly onto both minerals, rendering them highly hydrophobic. However, when Fe^2+^ was introduced alongside NaOL, the contact angle of calcite plummeted to 30.45° (Figure 4c), indicating that ferrous ions effectively prevented collector adsorption and restored the surface to a hydrophilic state. Conversely, the contact angle of rhodochrosite treated with Fe^2+^ and NaOL rose to 109.07°, indicating that Fe^2+^ did not inhibit NaOL adsorption but rather maintained the high hydrophobicity of the rhodochrosite. Collectively, these results demonstrate that Fe^2+^ significantly hinders the hydrophobization of calcite by NaOL while preserving the hydrophobic nature of rhodochrosite, findings which align well with the micro-flotation results.

### 2.3. Zeta Potential Measurements

**Figure 5:**
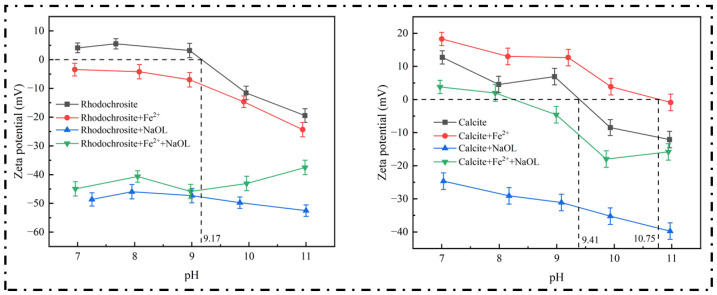
Electrokinetic potential measurements for the rhodochrosite and calcite with pH (NaOL: 1.3 × 10^−4^ mol/L, Fe^2+^: 2.5 × 10^−3^ mol/L).

Electrokinetic studies were performed to clarify how charged species adsorb onto mineral interfaces. In particular, we utilized this method to examine the distinct alterations in surface electrical characteristics of rhodochrosite and calcite caused by Fe^2+^ and oleate ions over a wide pH range. As depicted in Figure 5, the zeta potential profiles vary significantly with pH under different chemical environments. The measured isoelectric points (IEPs) were 9.17 for rhodochrosite and 9.41 for calcite, which aligns well with values cited in previous literature [24,25,26]. Introducing NaOL alone resulted in markedly more electronegative values for both rhodochrosite and calcite, pointing to the robust attachment of anionic collector molecules to the mineral interfaces. Conversely, the addition of Fe^2+^ elicited distinct electrokinetic responses from the two minerals. In contrast to the negligible variation observed for rhodochrosite, the zeta potential of calcite moved sharply towards the positive direction. This contrast indicates a markedly higher adsorption density of Fe^2+^ on calcite relative to rhodochrosite.

Notably, the zeta potential of rhodochrosite did not shift positively upon the addition of Fe^2+^; conversely, it trended toward more negative values. This behavior is primarily attributed to the rapid hydrolysis and oxidation of Fe^2+^ under alkaline conditions. Consequently, the generated Fe_x_(OH)_y_ species fail to form a stable, continuous adsorption layer on the rhodochrosite surface. Given the weak affinity between surface Mn^2+^ sites and iron species, Fe_x_(OH)_y_ deposits discontinuously, preventing the effective neutralization or masking of the intrinsic negative surface sites. These findings suggest that Fe^2+^ exerts a limited modulating effect on the interfacial electrical properties of rhodochrosite. Thus, rather than functioning as a non-selective depressant, the unstable adsorption of Fe^2+^ on rhodochrosite amplifies the electrokinetic contrast between it and calcite.

Upon the sequential addition of Fe^2+^ and NaOL, the zeta potential of rhodochrosite shifted sharply negatively, approaching the values observed with NaOL treatment alone. In contrast, the magnitude of the potential shift for calcite was significantly attenuated. Under pH 9.0 conditions, the zeta potential of rhodochrosite conditioned with Fe^2+^ and NaOL dropped to −45.86 mV, marking a significant reduction of 49.03 mV relative to the bare mineral (3.17 mV). Under identical conditions, calcite exhibited a decrease of only 11.52 mV. This discrepancy implies that calcite exhibits a preferential interaction with Fe^2+^, generating a surface film that significantly impedes the further uptake of the collector. Conversely, rhodochrosite maintains its capacity to adsorb oleate species due to the instability of the formed Fe^2+^ film.

The observed electrokinetic behaviors align well with the outcomes of the micro-flotation tests and wettability assessments. This consistency further validates the selective modulation mechanism of Fe^2+^ on the rhodochrosite-calcite system from an electrokinetic perspective.

### 2.4. SEM-EDS Test

To elucidate the microscopic mechanisms governing the differential flotation of calcite and rhodochrosite mediated by Fe^2+^, comparative analyses of the surface morphology and elemental composition were conducted via SEM-EDS. To visualize surface topography and reagent-induced alterations at the micron and nanometer levels, Scanning Electron Microscopy (SEM) was employed. This technique was integrated with Energy Dispersive Spectroscopy (EDS) for the purpose of qualitatively and semi-quantitatively assessing the elemental shifts resulting from Fe^2+^ adsorption.

**Figure 6:**
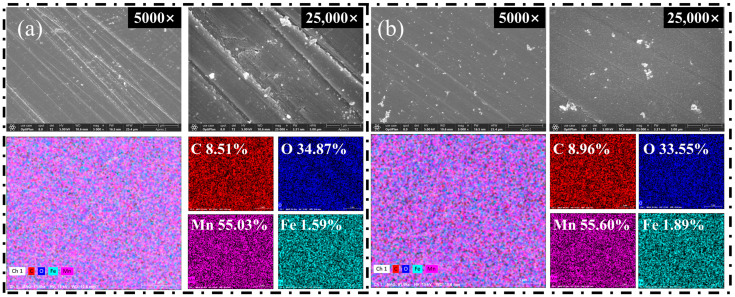
SEM-EDS images of rhodochrosite surfaces before (**a**) and after (**b**) Fe^2+^ treatment.

**Figure 7:**
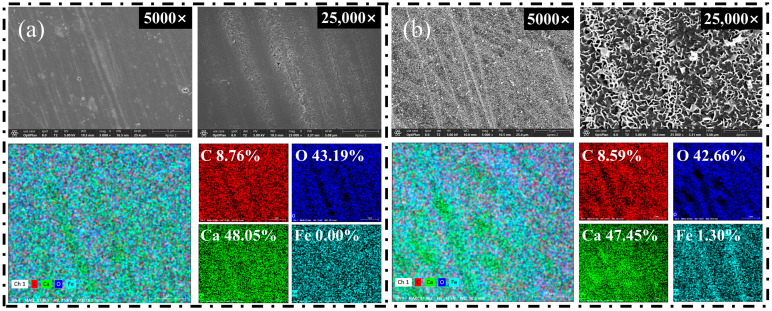
SEM-EDS images of calcite surfaces before (**a**) and after (**b**) Fe^2+^ treatment.

Figure 6 and Figure 7 depict the surface topography and chemical constituents of rhodochrosite and calcite. As shown in Figure 6a and Figure 7a, the untreated surfaces of both minerals exhibited relatively smooth, compact morphologies. The left panel displays the surface morphology at a magnification of 5000×, while the right panel illustrates the microstructure at 25,000× magnification. Mechanical grinding striations were clearly visible, with no significant secondary deposits or coating layers detected. EDS analysis indicated that the rhodochrosite surface was predominantly composed of Mn, O, and C, with an Fe content of only 1.59%, confirming the absence of significant iron-bearing contaminants. Similarly, the calcite surface consisted primarily of Ca, O, and C with negligible Fe content.

Upon Fe^2+^ treatment, the two minerals exhibited divergent surface characteristics. As illustrated in Figure 6b, the morphology of rhodochrosite showed negligible changes. The surface retained its smooth texture and mechanical striations, remaining devoid of observable flocculent precipitates or continuous coating layers. Quantitative EDS analysis revealed only minor variations in the concentrations of Mn, O, and C (shifting from 55.03%, 34.87%, and 8.51% to 55.60%, 33.55%, and 8.96%, respectively). Conversely, the Fe content rose marginally from 1.59% to 1.89%, indicating negligible surface enrichment. This suggests that Fe^2+^ adsorption on the Mn^2+^-dominated rhodochrosite surface is limited, preventing the formation of an effective hydrophilic passivation layer.

In contrast, the calcite surface underwent significant microstructural reconstruction. As illustrated in Figure 7b, numerous irregular flocculent structures emerged on the treated calcite surface, forming a porous yet distinct overlayer. EDS elemental mapping revealed a marked enhancement in the Fe signal, with the atomic percentage increasing from negligible levels to 1.30%. Concurrently, the relative concentration of Ca diminished from 48.05% to 47.45%. These findings suggest that substantial deposition of iron-bearing species occurred on the calcite surface, effectively masking the intrinsic Ca^2+^ active sites.

The findings suggest a strong affinity of Fe^2+^ ions for the calcite surface under the studied conditions. This selective enrichment is attributed to the strong affinity of Fe^2+^ hydrolysis products for surface Ca^2+^ sites. In alkaline flotation systems, Fe^2+^ readily hydrolyzes to form various iron hydroxy complexes. These species adhere to the calcite surface via adsorption or precipitation, gradually forming a strongly hydrophilic iron-bearing coating. This layer effectively shields the Ca^2+^ active sites, which are typically available for the chemical adsorption of anionic collectors such as sodium oleate. Consequently, this attenuates collector adsorption and diminishes surface hydrophobicity, resulting in the marked depression of calcite flotation. Furthermore, the observed flocculent structures suggest that iron-bearing species do not exist as isolated ions but rather as film-like aggregates covering the surface.

Based on the combined SEM morphology and EDS quantitative results, it is inferred that Fe^2+^ ions are unable to form a continuous or dense iron-bearing coating on the rhodochrosite surface. Given that the rhodochrosite surface is dominated by Mn^2+^ sites, its affinity for Fe^2+^ hydrolysis products is significantly lower compared to that of the Ca^2+^ sites on calcite. Consequently, the adsorption and deposition of Fe^2+^ species on rhodochrosite are severely restricted. Consequently, the surface binding centers of rhodochrosite were preserved despite the Fe^2+^ treatment, thereby facilitating efficient collector attachment.

In summary, Fe^2+^ treatment induces negligible changes to the surface morphology and elemental composition of rhodochrosite, leaving its flotation behavior essentially unaffected. This contrasts sharply with calcite, where Fe^2+^ forms an iron-bearing coating that significantly inhibits floatability. These observations offer microstructural proof validating the preferential action of Fe^2+^ during the beneficiation process for the two minerals.

### 2.5. XPS Analyses

X-ray photoelectron spectroscopy (XPS) serves as a robust method for elucidating the chemical valence and elemental abundance of mineral surfaces following interaction with reagents [27,28]. Consequently, the surface speciation and binding mechanism of Fe^2+^ were probed using XPS analysis to provide molecular-level insights.

Table 1 details the atomic relative concentrations and the corresponding shifts of elements on the surfaces of rhodochrosite and calcite before and after interaction with Fe^2+^ ions. The XPS survey results provide quantitative evidence for the differential adsorption behavior of ferrous ions on the two minerals. For the calcite system, the introduction of Fe^2+^ resulted in a substantial surface reconstruction: the Fe atomic concentration surged from 0% to 10.04%, accompanied by a sharp decline in Ca concentration from 15.03% to 8.70% (a decrease of 6.33%). In stark contrast, the rhodochrosite surface exhibited a much lower affinity for ferrous ions, with the Fe concentration increasing to only 4.01% and a negligible reduction in surface Mn sites (−1.31%). These results are intrinsically linked to the selective flotation mechanism investigated in this study. The significantly higher “Shift-C-value” (+10.04%) compared to the “Shift-R-value” (+4.01%) confirms that iron species preferentially adsorb onto or precipitate on the calcite surface. This extensive coverage of iron species effectively masks the active Ca sites on calcite, thereby hindering collector adsorption and resulting in its strong depression. Conversely, the limited iron coverage on rhodochrosite preserves its surface properties, allowing for the achievement of selective separation as proposed in the result of SEM-EDS.

**Figure 8:**
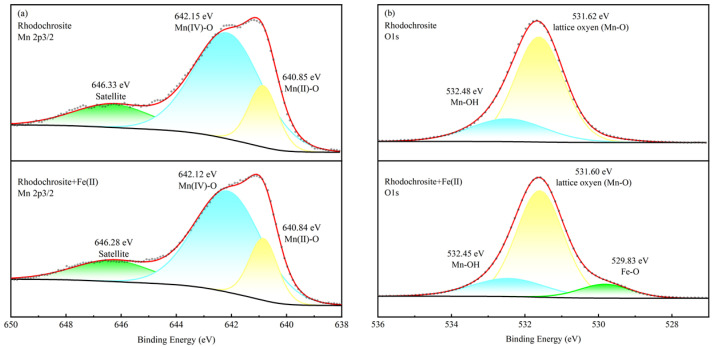
High-resolution photoemission peaks of Mn 2p and O 1s obtained from rhodochrosite in the absence and presence of ferrous ions. (**a**) Mn 2p3/2 of rhodochrosite before and after treatment. (**b**) O1s of rhodochrosite before and after treatment.

**Figure 9:**
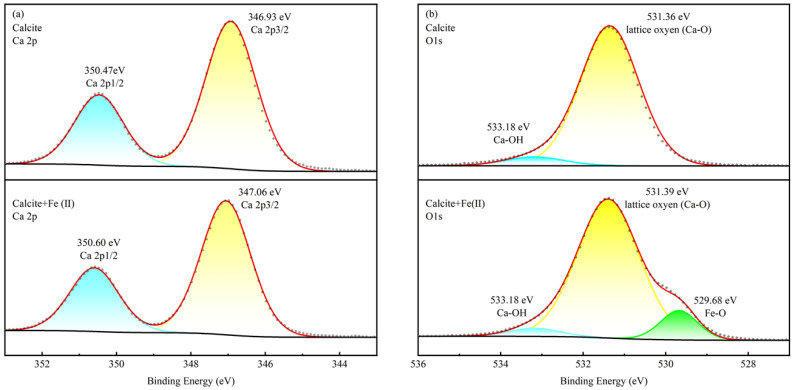
High-resolution photoemission peaks of Ca 2p and O 1s obtained from calcite in the absence and presence of ferrous ions. (**a**) Ca 2p of calcite before and after treatment. (**b**) O1s of calcite before and after treatment.

To further investigate the surface speciation, high-resolution XPS scans were performed. Figure 8 presents the fitted Mn 2p and O 1s spectra for rhodochrosite, while Figure 9 displays the Ca 2p and O 1s spectra for calcite.

The high-resolution Mn 2p3/2 spectral profile of rhodochrosite is depicted in Figure 8a. Deconvolution of the pure mineral spectrum reveals a triplet structure (three peaks). Specifically, the components located at binding energies of 640.85 eV and 642.15 eV are ascribed to the Mn(IV) oxidation state [29,30] and Mn(II) [25,31], respectively. The presence of multiple species is likely due to the partial hydration of the surface [32]. After the introduction of reagents, these binding energies showed only a negligible shift to 640.84 eV and 642.12 eV (dropping by 0.02 eV and 0.03 eV, respectively). This minimal variation suggests that the electronic state of surface Mn atoms was not significantly altered by Fe^2+^ adsorption. In the O 1s scan (Figure 8b), the component at 531.62 eV is identified as lattice oxygen (Mn-O-C) [33,34], whereas the peak at 532.48 eV corresponds to surface hydroxyls (Mn-OH) [29,35]. Notably, conditioning with Fe^2+^ resulted in the emergence of a new signal at 529.83 eV, which is ascribed to Fe-O bonding [36,37,38].

The Ca 2p core-level spectra are presented in Figure 9a. For the bare surface, characteristic peaks were recorded at 346.93 eV (2p3/2) and 350.47 eV (2p1/2) [39,40,41]. Upon interaction with Fe^2+^, a uniform positive shift of 0.13 eV was detected, moving the peaks to 347.06 eV and 350.60 eV. This substantial variation implies that the reagents interact more strongly with Ca sites than with Mn sites [42]. In the O 1s spectra, Figure 9b, peaks at 531.36 eV and 533.18 eV correspond to Ca-O-C [43,44,45] and Ca-OH groups [18,20,41,46,47], respectively. Besides the typical peaks for lattice oxygen and surface hydroxyls, a distinct new peak emerged at 529.68 eV. This feature corresponds to the Fe-O bond, providing solid evidence for the formation of iron hydroxide precipitates.

**Figure 10:**
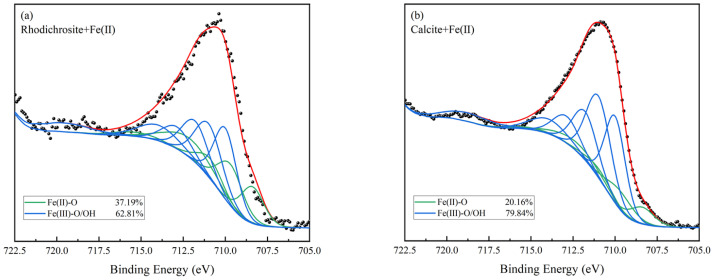
XPS multiplet fitting analysis of Fe 2p spectra for rhodochrosite (**a**) and calcite (**b**) treated with Fe^2+^.

Multiplet fitting analysis was conducted on the high-resolution Fe 2p spectra of both minerals (Figure 10). On the treated rhodochrosite, the Fe(II)-O species comprises 37.19% of the total intensity, while Fe(III)-O/OH species account for 62.81%. Conversely, on treated calcite, the Fe(II)-O fraction is only 20.16%, whereas the Fe(III)-O/OH proportion rises to 79.84%. Notably, the proportion of surface Fe(III) species on calcite is 17.03% higher than on rhodochrosite. These findings align with both the ToF-SIMS analysis and zeta potential measurements.

In summary, while Fe^2+^ adsorbs onto both minerals, the interaction is markedly stronger and the oxidation extent greater on the calcite surface. This disparity stems from the partial ion exchange of Fe^2+^ with Mn^2+^ in rhodochrosite, leading to the incorporation of iron into the lattice. Consequently, the resulting hydrophilic layer on rhodochrosite is discontinuous, exerting a negligible impact on flotation performance. In contrast, the robust adsorption on calcite facilitates the formation of a dense, continuous hydrophilic layer, significantly depressing its floatability.

### 2.6. ToF-SIMS Tests

Time-of-Flight Secondary Ion Mass Spectrometry (ToF-SIMS) is extensively utilized in mineral processing research due to its superior sensitivity in analyzing surface chemical compositions. By identifying characteristic ion fragments, this technique elucidates the interaction mechanisms between flotation reagents and mineral surfaces [48,49].

**Figure 11:**
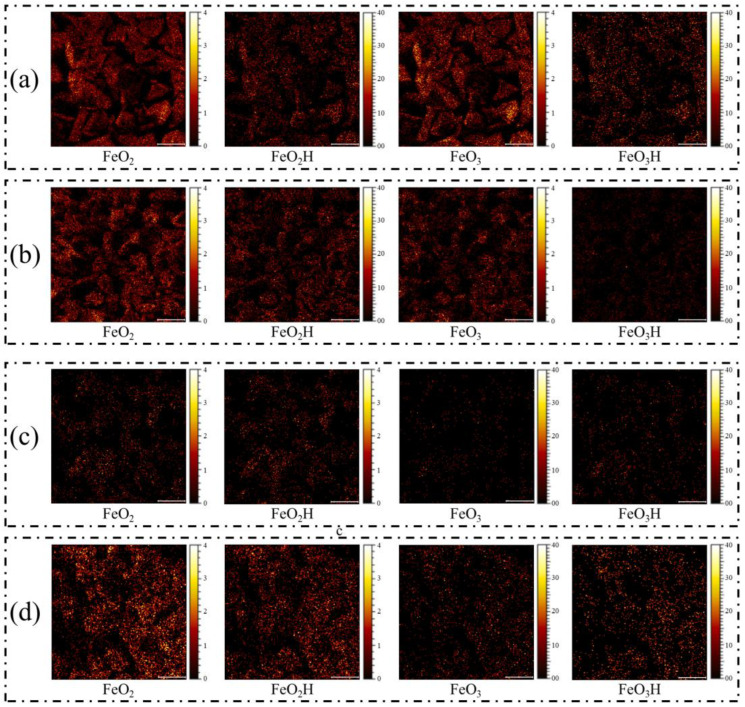
ToF-SIMS ion images of rhodochrosite surface (**a**); ToF-SIMS ion images of rhodochrosite surface after Fe^2+^ treatment (**b**); ToF-SIMS ion images of calcite surface (**c**); ToF-SIMS ion images of calcite surface after Fe^2+^ treatment (**d**).

Figure 11 presents the 2D mapping of representative iron-oxygen fragments (FeO_2_, FeO_2_H, FeO_3_, and FeO_3_H) on the rhodochrosite surface. As shown in Figure 11a, the bare rhodochrosite exhibits scattered, low-intensity background signals of iron species, likely originating from trace lattice impurities. Significantly, after conditioning with Fe^2+^ (Figure 11b), the surface morphology and ion intensity maps show negligible changes compared to the untreated sample. Unlike the dense coverage observed on calcite, the iron species on the rhodochrosite surface remain sparse and discontinuous, with no widespread accumulation or formation of a uniform coating layer. This visual evidence corroborates the XPS and adsorption data, confirming that ferrous ions do not undergo significant chemisorption or precipitation on the rhodochrosite surface, thereby preserving its floatability.

Previous studies indicate that, under appropriate conditions, Fe^2+^ can undergo partial isomorphous substitution with Mn^2+^ within the rhodochrosite crystal lattice [50,51]. This phenomenon is attributed to the similar ionic radii of the two divalent cations (Mn^2+^ ≈ 97 pm and Fe^2+^ ≈ 92 pm) [52]. Consequently, the surface reconstruction involving Fe^2+^ leads to the partial rupture of Mn-Fe covalent bonds and the formation of new species such as Fe_x_(OH)_y_ or Mn-Fe-O/OH. However, as this resulting layer is porous and discontinuous, abundant Mn^2+^ active sites remain exposed, thereby permitting substantial collector adsorption.

In stark contrast to the observations on rhodochrosite, the ToF-SIMS ion images reveal a significant accumulation of iron species on the calcite surface after treatment. Figure 11c displays the distribution of representative iron-oxygen fragments (FeO_2_, FeO_2_H, FeO_3_, and FeO_3_H) on the untreated calcite surface. The bare calcite surface shows only negligible, low-intensity background signals, confirming a clean baseline. However, after interaction with ferrous ions (Fe^2+^), a dramatic change is observed in Figure 11d. The mapping images exhibit a high density of bright spots corresponding to iron species, indicating a widespread and uniform adsorption of iron hydroxides across the calcite particles. The intensity of fragments such as FeO_2_ and FeO_2_H is markedly enhanced, suggesting the formation of a dense iron-containing passivation layer. This extensive surface coverage serves as a hydrophilic barrier, effectively blocking the adsorption of the collector and thereby depressing the flotation of calcite. The ToF-SIMS findings corroborate the morphological characterization by SEM-EDS and are consistent with the surface elemental concentrations determined by XPS.

**Figure 12:**
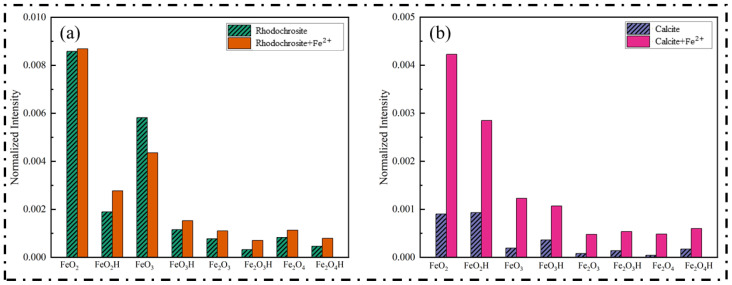
The normalized intensities of characteristic iron-oxygen fragments on rhodochrosite (**a**) and calcite (**b**) surfaces.

To provide a semi-quantitative verification of the surface adsorption behavior, the normalized intensities of characteristic iron-oxygen fragments were extracted from the ToF-SIMS spectra and summarized in Figure 12.

As illustrated in Figure 12b, the untreated calcite surface exhibits extremely low baseline intensities for all iron species. However, upon treatment with ferrous ions, a dramatic surge in signal intensity is observed. This sharp elevation confirms the substantial accumulation and chemisorption of iron species, consistent with the formation of a dense hydroxide coating. In distinct contrast, the results for rhodochrosite in Figure 12a show a completely different trend. While the bare rhodochrosite exhibits a higher inherent iron baseline (likely due to lattice impurities), the addition of ferrous ions did not induce any significant increase in surface iron intensity. The signal levels for the treated sample remain comparable to those of the fresh ore, with only negligible fluctuations. This quantitative comparison clearly demonstrates that ferrous ions do not effectively adsorb onto the rhodochrosite surface, further validating the high selectivity of the depression mechanism.

### 2.7. Visual MINTEQ Calculation

**Figure 13:**
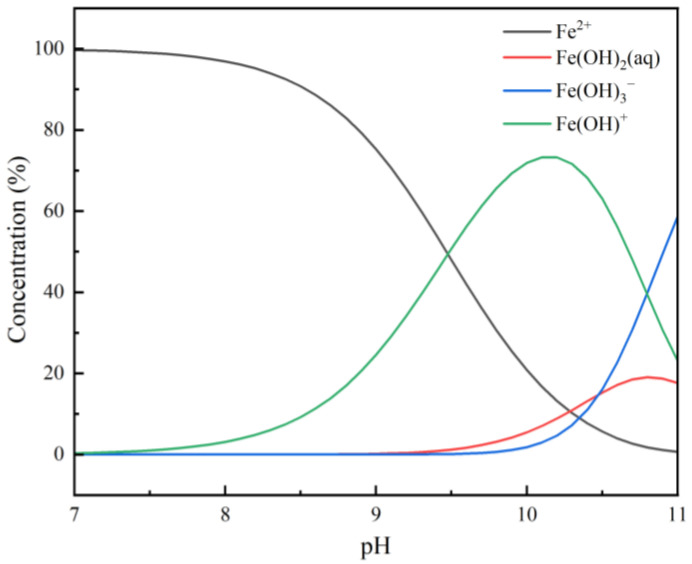
Solution chemistry and speciation of Fe^2+^ under varying pH conditions.

To elucidate the behavior of Fe^2+^ across varying pH conditions, the solution species distribution was simulated using Visual MINTEQ (Figure 13). The results indicate that between pH 7.0 and 8.0, aqueous iron exists predominantly as free Fe^2+^. As pH increases, the concentration of free Fe^2+^ declines, concomitant with the emergence of hydrolyzed species such as Fe(OH)^+^ and Fe(OH)_2_(aq). In the pH 9.5~10.0 range, Fe(OH)^+^ becomes the dominant species, alongside significant increases in Fe(OH)_2_(aq) and Fe(OH)_3_^−^, confirming the strong propensity of Fe^2+^ for hydrolysis and precipitation under alkaline conditions.

Correlating these findings with micro-flotation results, it is evident that Fe^2+^ hydrolysis products formed at alkaline pH are the primary drivers of the altered calcite flotation behavior. These surface-active hydrolyzed species readily adsorb onto or precipitate at the Ca^2+^ sites on the calcite lattice. Furthermore, as hydrolysis progresses, the accumulation of iron hydroxy complexes hinders collector adsorption via steric and electrostatic blockage. This mechanism corroborates the SEM-EDS and XPS observations, specifically the marked surface enrichment of Fe and the masking of active Ca sites on calcite.

In contrast, despite undergoing identical solution hydrolysis, the resulting products exhibit a negligible affinity for the rhodochrosite surface. Even at pH levels dominated by Fe(OH)^+^ and Fe(OH)_2_(aq), rhodochrosite recovery remains robust, indicating that these species fail to form a stable adsorption layer on the Mn^2+^-dominated surface. This aligns with the spectroscopic evidence (SEM–EDS and XPS), which confirmed negligible surface Fe uptake and the preservation of Mn active sites.

In summary, the pH-dependent hydrolysis and species distribution of Fe^2+^ provide the fundamental chemical basis for the differential flotation behavior of the two minerals. Specifically, alkaline hydrolysis products preferentially passivate the calcite surface while exhibiting minimal interaction with rhodochrosite, thereby facilitating the selective depression of calcite.

**Figure 14:**
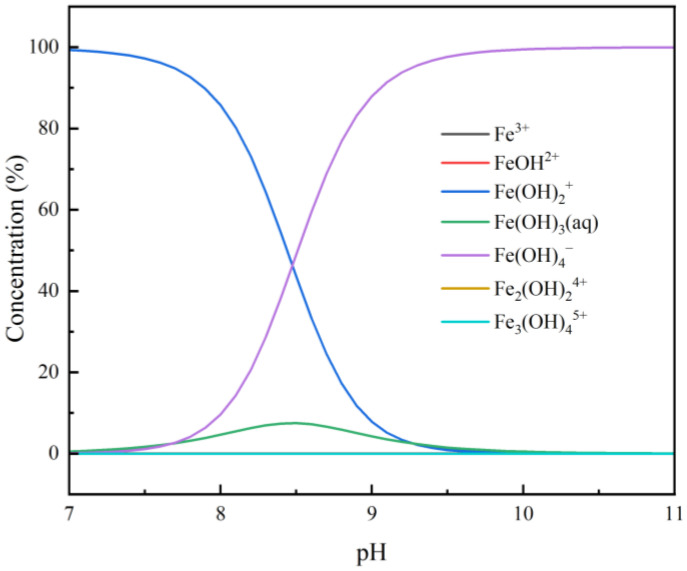
Solution chemistry and speciation of Fe^3+^ under varying pH conditions.

Considering the oxidative environment inherent to the flotation process, the potential transformation of ferrous ions (Fe^2+^) to ferric ions (Fe^3+^) was investigated. The speciation of Fe^3+^ within the pH range of 7.0~11.0 was simulated to evaluate its impact on the flotation system (Figure 14).

The simulation reveals a significant shift in hydrolysis products: the cationic species Fe(OH)_2_^+^ predominates at pH < 8.5, while the anionic species Fe(OH)_4_^−^ becomes dominant at pH > 8.5. Crucially, at pH 9.0—identified in our flotation tests as the optimal condition for separating calcite from rhodochrosite—the concentration of Fe(OH)_4_^−^ approaches its maximum.

This correlation suggests that if Fe^2+^ oxidation occurs, the resulting Fe(OH)_4_^−^ species plays a pivotal role at the separation pH. Being anionic and highly hydrophilic, Fe(OH)_4_^−^ can strongly adsorb onto the positively charged sites of the mineral surface or form a hydration layer, thereby inhibiting collector adsorption and enhancing the depression of calcite. This provides a thermodynamic basis for the effective separation observed at pH 9.0.

## 3. Discussion

### 3.1. Correlation Between Solution Chemistry and Selective Flotation

The selective separation of semi-soluble salt minerals, such as calcite and rhodochrosite, is fundamentally governed by the interplay between mineral surface dissolution and the hydrolysis of metal ions. The flotation results indicated that pH 9.0 is the critical window for achieving selective separation, where Fe^2+^ significantly depresses calcite (recovery < 20%) while barely affecting rhodochrosite (recovery > 75%).

According to the Visual MINTEQ simulation, at pH 9.0, the dominant iron species shifts from free Fe^2+^ to hydrolyzed species, particularly Fe(OH)^+^ and the precipitation of Fe(OH)_2_(s). It is well-established that metal hydroxides exhibit strong depression effects due to their inherent hydrophilicity. The discrepancy in flotation recovery suggests that the interaction affinity of these hydrolyzed iron species differs significantly between the calcite and rhodochrosite surfaces. The presence of Fe^2+^ creates a distinct physicochemical environment, likely driven by the thermodynamic stability of the surface precipitates relative to the bulk solution.

### 3.2. Surface Adsorption Mechanism and Layer Characteristics

**Figure 15:**
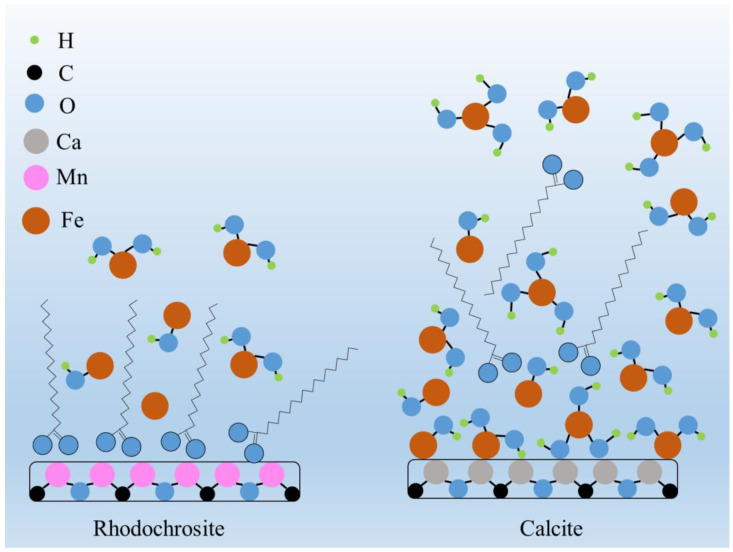
Mechanism diagram of Fe^2+^ on rhodochrosite and calcite surface.

The core of the selective depression mechanism lies in the chemisorption behavior and the subsequent formation of surface layers, as elucidated by surface analysis techniques. The schematic mechanism of ferrous ion adsorption on the surfaces of rhodochrosite and calcite is illustrated in Figure 15.

On Calcite Surfaces: The XPS and ToF-SIMS results provide compelling evidence of a strong chemical interaction. The high atomic concentration of iron detected on the calcite surface indicates that Fe^2+^ (and its hydrolysis products) chemically adsorbs onto the carbonate sites. This chemisorption serves as a nucleation template, promoting the rapid growth of a dense and uniform iron hydroxide layer, as visualized by SEM-EDS. This layer effectively “masks” the original surface properties of calcite.

On Rhodochrosite Surfaces: In contrast, the iron species show a much weaker affinity for the rhodochrosite lattice. The SEM and XPS data revealed only a thin, discontinuous distribution of iron species. This suggests that the lattice compatibility or chemical bonding energy between Fe^2+^ species and the MnCO_3_ surface is insufficient to support the formation of a continuous passivation layer. Consequently, a significant portion of the rhodochrosite surface remains exposed and active.

### 3.3. Modulation of Wettability and Collector Interaction

The alteration of surface morphology directly dictates the mineral’s wettability and its interaction with the collector. The zeta potential measurements demonstrated a significant shift in the surface charge of calcite after Fe^2+^ treatment, confirming the extensive coverage of electrokinetically active iron hydroxides.

This surface modification leads to the “Hydrophilic Barrier Effect.” As confirmed by contact angle measurements, the dense iron hydroxide layer on calcite renders the surface highly hydrophilic. Mechanistically, this layer acts as a physical and electrostatic barrier that inhibits the adsorption of the collector. The collector molecules cannot penetrate this dense hydration shell to reach the specific adsorption sites on the calcite surface. Conversely, due to the discontinuous nature of the iron layer on rhodochrosite, the collector molecules can still access and adsorb onto the exposed MnCO_3_ sites, thereby maintaining sufficient hydrophobicity for bubble attachment.

### 3.4. Surface Adsorption Versus Bulk Collector Depletion

A critical concern in using metal ions as depressants is the potential consumption of the collector through bulk precipitation or complexation. Previous molecular dynamics studies have indicated that iron species (e.g., Fe(OH)^2+^ and Fe(OH)_2_^+^) often exhibit a higher binding energy with oleate anions (approx. −1.2 eV) than with mineral surfaces (approx. −0.5 eV), leading to the formation of stable iron-oleate complexes in solution [53]. If this mechanism were dominant in our system, the depletion of the collector would result in the non-selective depression of both calcite and rhodochrosite.

However, the flotation results in this study demonstrate a stark contrast: while calcite is strongly depressed (recovery < 20%), rhodochrosite maintains a high recovery (>75%). This high selectivity implies that the bulk concentration of the collector is not significantly exhausted by ferrous ions. This can be explained by the solution chemistry at pH 9.0, where ferrous ions predominantly hydrolyze to form neutral, insoluble Fe(OH)_2_ (or Fe(OH)_3_ upon oxidation) precipitates. Instead of consuming the collector in the bulk solution, these hydrophilic species selectively deposit on the calcite surface (as confirmed by the high Fe atomic concentration in XPS analysis), creating a physical barrier that hinders collector adsorption. Thus, the depression mechanism is governed by selective surface passivation rather than bulk reagent depletion.

### 3.5. Implications for Refractory Carbonate Separation

This study highlights the importance of selective interfacial regulation. Unlike traditional depressants that may rely on bulk precipitation, the role of Fe^2+^ here is highly surface-specific. The intrinsic difference in the adsorption capacity of ferrous ions on calcium versus manganese carbonate surfaces provides a robust theoretical basis for manipulating surface properties. This approach offers a promising pathway for the flotation separation of complex, refractory carbonate ores where traditional depressants fail to achieve high selectivity.

### 3.6. Limitations and Future Work

It is worth noting that this study primarily focuses on the fundamental surface chemistry and the intrinsic interaction mechanisms between ferrous ions and carbonate minerals using pure mineral samples. While single mineral tests provide a pristine environment to characterize surface modifications (as evidenced by XPS and ToF-SIMS), practical flotation systems are inherently more complex. In mixed mineral systems or real ores, factors such as dissolved ion interference (e.g., competitive adsorption between Ca^2+^ and Fe^2+^) and heterocoagulation (slime coating of fine calcite on rhodochrosite) may affect the selectivity. Furthermore, the degree of liberation is critical for real ores. For composite particles containing both calcite and rhodochrosite, the preferential formation of a hydrophilic iron hydroxide layer on the calcite regions would likely depress the entire composite particle into the tailings. This mechanism suggests that while ferrous ions can effectively improve concentrate grade, adequate grinding to ensure high liberation is a prerequisite for optimal recovery. Future work will focus on optimizing this reagent regime in binary mixed systems and bench-scale real ore flotation to address these practical challenges.

## 4. Materials and Methods

### 4.1. Materials and Reagents

**Figure 16:**
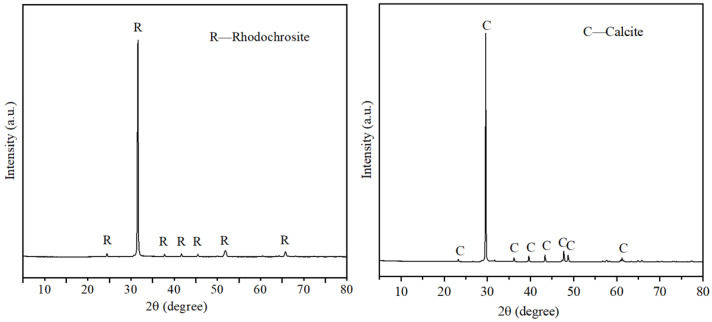
XRD pattern of rhodochrosite and calcite.

Rhodochrosite and calcite samples were sourced from Guangdong and Yunnan Provinces, respectively. The multi-element analysis results of the raw materials are listed in Table 2, whereas Figure 16 depicts the corresponding X-ray diffraction (XRD) profiles. The purity of rhodochrosite is 95.01%, and the purity of calcite is 96.45%. These analyses confirm that the purity of both minerals satisfies the stringent requirements for pure mineral investigations. Analytical grade (AR) chemicals were utilized for all experiments. Ferrous chloride tetrahydrate (FeCl_2_ 4H_2_O) and sodium carbonate Na_2_CO_3_, used as a pH regulator) were sourced from Xi’an Tianmao Baoding Biotechnology Co., Ltd (Xi’an, China). Hydrochloric acid (HCl), employed for pH adjustment, was obtained from Guangzhou Hewei Pharmaceutical Technology Co., Ltd (Guangzhou, China). Sodium oleate (NaOL), serving as the collector, was purchased from Shanghai Macklin Biochemical Technology Co., Ltd (Shanghai, China). Throughout the experimental work, including both flotation trials and analytical measurements, ultrapure water (resistivity: 18.25 MΩ·cm) was utilized.

### 4.2. Micro-Flotation Experiments

Micro-scale flotation experiments were conducted using an XFG II5-35 machine (40 mL volume) at an agitation speed of 1700 rpm. For each test, 2.0 g of mineral powder (particle fraction: 38~74 μm) was mixed with 40 mL ultrapure water and conditioned for 60 s. This was followed by the introduction of the FeCl_2_ solution, which was conditioned for 3 min. The slurry pH was regulated via dilute HCl or Na_2_CO_3_, followed by 60 s of conditioning. Upon adding the collector and stirring for another 3 min, the froth was collected for 4 min. After adding the collector and conditioning for a further 3 min, froth collection was performed for 4 min. Finally, the flotation recovery was determined using the dry mass of the concentrate and tailings. All experiments were performed in triplicate, and the average values were reported.

### 4.3. Contact Angle Measurements

Contact angle measurements were conducted using the sessile drop method on a JY-82C goniometer (Chengde Dingsheng, Chengde, China) to assess surface hydrophobicity. The procedure involved suspending 2.0 g of mineral powder (38~74 μm) in 40 mL of deionized water. The reagent conditioning protocol mirrored that of the flotation experiments. Subsequently, the mineral particles underwent filtration, drying, and compression into tablets. A water droplet was dispensed onto the tablet via a microsyringe. Upon reaching equilibrium, the contact angle was determined by the instrument’s software. The reported value is the average of three independent measurements.

### 4.4. Zeta Potential Measurements

A Zetasizer Pro (Malvern Panalytical, Malvern, UK) was utilized to determine the electrokinetic potential. Prior to analysis, minerals were comminuted to a size of −5 μm. A stock suspension was created by dispersing 40 mg of solids into 40 mL of water. After stirring for 1 min, the slurry underwent conditioning with reagents following the flotation protocol. Following 10 min of settling, the supernatant was collected. The reported zeta potential represents the mean of three independent readings.

### 4.5. SEM-EDS Tests

To investigate surface textures and chemical composition, we utilized SEM imaging coupled with EDS analysis. The specific instrument used was a Thermo Scientific Apreo 2 S equipped with a Bruker X Flash 6 sensor (Thermo Scientific, Waltham, MA, USA). The specimens were prepared by sectioning the minerals into rectangular prisms with dimensions of 10 mm × 10 mm × 5 mm. These blocks were polished to a mirror finish and ultrasonically cleaned to eliminate surface contaminants, thereby ensuring high-quality imaging. Surface conditioning was performed in a 40 mL cell of an XFG II 5-35 laboratory flotation apparatus. The specimen was immersed in 40 mL of deionized water under an agitation speed of 800 rpm. Subsequently, it was treated with FeCl_2_ solution, and the pH was regulated to 9.0 using Na_2_CO_3_ or HCl. All reagent dosages and conditioning intervals were maintained consistent with the flotation experiments. Following conditioning, the block was extracted using forceps, dried in ambient air, and the specific surface targeted for analysis was marked. To ensure the representativeness of the results, spectral data were collected from at least three different locations on each sample surface.

### 4.6. XPS Analyses

For XPS characterization, 2.0 g of the −38 μm size fraction was agitated in a beaker using a temperature-controlled magnetic stirrer. Reagents were introduced following the exact conditions of the flotation tests. The resulting solids were filtered, dried, and subsequently pressed into tablet form. The spectra were acquired using a Thermo Scientific K-Alpha spectrometer (Thermo Scientific, Waltham, MA, USA) utilizing monochromatized Al Kα radiation.

### 4.7. ToF-SIMS Tests

Prior to spectral analysis, mineral samples were prepared to mimic flotation conditions. A suspension containing 2.0 g of minerals (−74 + 38 μm) and 40 mL of water was treated with FeCl_2_, with the pH adjusted to 9.0. Reagent contact times and dosages matched the flotation tests exactly. The filtered and air-dried samples were subsequently characterized using a PHI nano TOF II instrument. The device employed a 30 keV Bi3+ beam to scan a 100 × 100 μm^2^ area (512 × 512 pixels) in high-mass-resolution mode.

### 4.8. Visual MINTEQ Calculation

Visual MINTEQ is a chemical equilibrium model widely utilized to predict ionic speciation, solubility limits, and solid-phase precipitation in aqueous systems. In this study, Visual MINTEQ^®^ 4.0 was employed to simulate the equilibrium speciation and solubility of Fe^2+^ across a range of pH conditions. Simulations were conducted at 25 °C with an initial Fe^2+^ concentration of 2.5 × 10^−3^ mol/L within a pH range of 7.0~11.0. These simulation outcomes establish a solid theoretical basis for understanding the mode of action through which ferrous ions alter the interfacial attributes of both carbonate minerals.

## 5. Conclusions

Through a comprehensive suite of analytical techniques—ranging from micro-flotation and wettability assessments to advanced surface characterizations like ToF-SIMS, XPS, and SEM-EDS—this work has elucidated the selective depression of calcite by ferrous ions. Combined with solution chemistry modeling, the study’s primary findings are outlined below:

(1) Selective Depression Performance: Micro-flotation and contact angle measurements confirmed that Fe^2+^ acts as a selective depressant for calcite while exerting negligible influence on rhodochrosite recovery. Notably, in the presence of Fe^2+^ and NaOL, rhodochrosite maintained a high degree of hydrophobicity (contact angle 109.07°), whereas calcite became highly hydrophilic (30.45°), enabling effective separation.
(2) Electrokinetic Mechanism: Zeta potential analysis revealed distinct electrokinetic responses. Fe^2+^ induced a significant positive shift in the surface charge of calcite, indicating robust adsorption. Conversely, rhodochrosite exhibited only a minor potential shift. Upon the subsequent addition of NaOL, the negligible potential change for calcite indicated that the pre-adsorbed Fe^2+^ layer effectively hindered the adsorption of the anionic collector.
(3) Surface Microstructure and Passivation: ToF-SIMS and SEM-EDS characterizations provided direct evidence of selective surface modification. Fe^2+^ formed a dense, continuous passivation layer composed of iron hydroxides (e.g., FeO_2_, FeO_2_H) and flocculent precipitates on the calcite surface, effectively masking the active Ca sites. In contrast, adsorption on rhodochrosite was patchy and discontinuous, leaving the Mn^2+^ active sites exposed for collector attachment.
(4) Surface Speciation: XPS results corroborated that the adsorption density and oxidation state of iron species are significantly higher on calcite than on rhodochrosite. The formation of a stable, high-coverage Fe(III)-dominant hydroxide film on calcite constitutes the primary physicochemical barrier to flotation.
(5) Solution Chemistry Basis: Thermodynamic simulations identified alkaline hydrolysis products as the active species responsible for depression. These species preferentially adsorb onto calcite due to a strong chemical affinity for surface Ca sites, whereas their interaction with the Mn-dominated rhodochrosite surface is limited, preventing the formation of a stable obstructing layer.

## Figures and Tables

**Table 1:** Atomic relative concentrations and the shifts of elements in the samples.

Sample		Element (Atomic %)			
	C 1s	Ca 2p	O 1s	Mn 2p	Fe 2p
Rhodochrosite	38.31	-	50.71	10.98	-
Rhodochrosite + Fe^2+^	35.95	-	50.37	9.67	4.01
Shift-R-value	−2.36	-	−0.34	−1.31	+4.01
Calcite	33.06	15.03	51.91	-	-
Calcite + Fe^2+^	30.18	8.70	51.08	-	10.04
Shift-C-value	−2.88	−6.33	−0.83	-	+10.04

**Table 2:** Chemical constituents of the rhodochrosite and calcite samples (wt%).

Sample	MnO	CaO	MgO	SiO_2_	TFe	Al_2_O_3_
Rhodochrosite	58.65	0.26	0.31	1.99	1.51	0.10
Calcite	0.06	54.01	0.22	2.05	0.62	0.11

## Data Availability

Data are contained within the article.
